# The advantages of UK Biobank's open‐access strategy for health research

**DOI:** 10.1111/joim.12955

**Published:** 2019-08-02

**Authors:** M. Conroy, J. Sellors, M. Effingham, T. J. Littlejohns, C. Boultwood, L. Gillions, C. L. M. Sudlow, R. Collins, N. E. Allen

**Affiliations:** ^1^ UK Biobank Cheadle Stockport UK; ^2^ Clinical Trial Service Unit and Epidemiological Studies Unit, Nuffield Department of Population Health University of Oxford Oxford UK; ^3^ Usher Institute of Population Health Sciences and Informatics University of Edinburgh Edinburgh UK

**Keywords:** epidemiology, public health, science

## Abstract

Ready access to health research studies is becoming more important as researchers, and their funders, seek to maximize the opportunities for scientific innovation and health improvements. Large‐scale population‐based prospective studies are particularly useful for multidisciplinary research into the causes, treatment and prevention of many different diseases. UK Biobank has been established as an open‐access resource for public health research, with the intention of making the data as widely available as possible in an equitable and transparent manner. Access to UK Biobank's unique breadth of phenotypic and genetic data has attracted researchers worldwide from across academia and industry. As a consequence, it has enabled scientists to perform world‐leading collaborative research. Moreover, open access to an already deeply characterized cohort has encouraged both public and private sector investment in further enhancements to make UK Biobank an unparalleled resource for public health research and an exemplar for the development of open‐access approaches for other studies.

## Graphical abstract

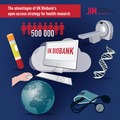

## Introduction

Over the last few decades, several large‐scale observational studies have been established to enable epidemiological research into the causes of the major diseases of middle and old age. Many of these studies express a commitment to open data sharing in order to facilitate research efforts, whilst ensuring appropriate commitment to participant confidentiality, consent and data protection regulations. This has become even more important in the era of genomics where meta‐analyses of data from multiple (largely retrospective) studies are essential to achieve the numbers required to perform population‐based genetic research 1, 2 and often requires collaboration with the team that set up the study. However, few epidemiological studies have been designed from the outset to be an open‐access resource available to academic and commercial researchers alike from around the world, with no preferential access.

This article describes the access policy of UK Biobank, how it has developed over time in relation to both the use of data and biological samples, and how it has facilitated collaborative research whereby the results can be shared by all.

## UK Biobank

UK Biobank is a large, prospective cohort study of 500 000 participants aged 40–69 years at the time of their baseline assessment visit during 2006–2010. The study was established to enable research into the lifestyle, environmental and genomic determinants of life‐threatening and disabling diseases of middle and old age. A vast amount of data was collected at recruitment, including self‐reported lifestyle and medical information (supplemented subsequently by antecedent information from health records), a wide range of physical measures (e.g. blood pressure, anthropometry, spirometry) and biological samples (blood, urine and saliva), of which further details are provided elsewhere 3. All of the data can be viewed on UK Biobank's online Data Showcase, including summary statistics for each data field available for research 4.

Since recruitment, UK Biobank has continued to be enhanced by converting the information contained in the biological samples, which are limited and depletable, into data that can be widely shared. This has included cohort‐wide genotyping (with subsequent imputation to over 90 million variants) 5 and whole exome sequencing, making it one of the largest studies in the world with detailed data on genetics, lifestyle and health outcomes. A range of blood and urine biomarkers of interest for research into common conditions (such as cardiovascular disease, cancer and diabetes) are also available for all 500 000 participants 6. UK Biobank continues to collect extensive data directly from participants. This includes a series of web‐based questionnaires sent to all participants with an email address (*n* = 330 000) about particular exposures (e.g. diet, occupation) and conditions (e.g. cognition, mental health, pain), objective physical activity monitoring (100 000), and ongoing assessments of multi‐modal imaging (target of 100,000) and cardiac monitoring (target of >20 000).

**Figure 1:**
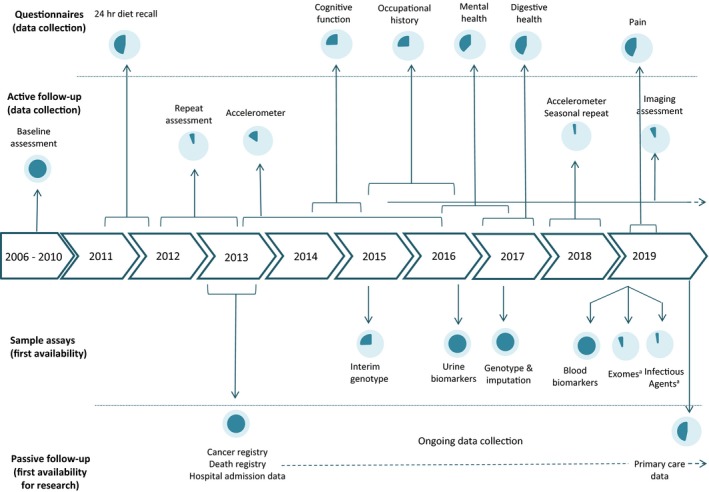
Timeline of data collection and availability for UK Biobank participants by mid‐2019. Pie chart indicates the proportion of the cohort that each data item is available for. ^a^Data on exome sequencing data (for 50 000 participants) and serological markers of infectious agents (for 10 000 participants) were made available in March 2019, with the intention to assay all 500 000 participants over the next few years.

As UK Biobank is a prospective study, considerable efforts are spent in following the health of all participants through linkage to electronic health datasets, including death and cancer registries, and primary and secondary care records (Fig 1). Several thousand incident cases of the most common conditions have already been identified, with many more cases expected to accrue over the next few years (Table 1). Efforts are underway to generate algorithmically derived health outcomes in order to facilitate a wide range of research using standardized outcome variables 7.

**Table 1:** Observed and expected numbers of selected health outcomes in UK Biobank over time^a^

Condition	Incident cases observed by 2016^a^	Incident cases predicted by 2026^b^
Dementia	4300	43 400
Stroke	7100	28 400
Myocardial infarction	8000	22 000
Chronic obstructive pulmonary disease	17 600	55 000
Parkinson's disease	2000	9700
Breast cancer	7000	18 000
Prostate cancer	6700	26 800
Colorectal cancer	4000	16 000

^a^Based on linkage to hospital inpatient records, death certificates, cancer registries and primary care (the latter extrapolated to the full cohort) up until 01 Jan 2016. ^b^Predicted numbers of cases were derived by applying ratios from a previous modelling exercise conducted for UK Biobank 31, which was based on UK age‐specific disease incidence rates, adjusted to take account of the numbers of disease cases observed so far in UK Biobank participants (who have lower rates of most diseases compared with similar aged people in the general UK population) in linked healthcare data from primary and secondary care sources.

## Access to the resource

UK Biobank was set up on the basis of a clear intention from its two core funders (the Medical Research Council and Wellcome Trust) as a de‐facto open‐access resource, with the aim to make the data as widely available as possible, with an equitable and transparent access policy 8.

In order to apply for access to data from UK Biobank, each applicant must demonstrate that they are a *bona fide* researcher (i.e. they must register from, and be affiliated with, an approved research institute) and the application must involve health‐related research that is in the public interest. All applicants are treated the same – whether academic, governmental, charitable or commercial, or whether from domestic or international organizations – and all applications are assessed according to the same consistent criteria.

All access applications are discussed and approved by the Access Sub‐Committee (ASC) of the UK Biobank Board. Access to data is relatively permissive, and review by the ASC seeks only to ensure that the research is viable and meets the requirements. The ASC's main responsibility is making strategic access decisions, particularly regarding contentious matters and the use of biological samples. Ethics advice is provided to the ASC on an independent consultancy basis by Oxford University's Ethox group 9.

Lay summaries of each approved application are published on the website. A standard material transfer agreement (MTA) is signed prior to any data delivery and governs how a researcher can use the data. All researchers must publish (or otherwise make publicly available) the findings of their research and return any derived data fields, and the methods used to generate them, back to UK Biobank. These data are available to other registered researchers, thereby encouraging transparency and reproducibility in scientific methods.

UK Biobank is established as a charity with access charges (reviewed on a periodic basis) which are set at a level that covers the costs of managing the access application process. In order to encourage use by potentially disadvantaged researchers, fees are subsidized for research groups from low and low‐to‐middle‐income countries (assessed according to the current World Bank guidelines) and for student projects.

## Evolution of UK Biobank's access approach

When the UK Biobank resource opened to researchers in April 2012, a relatively cautious approach to data access was taken. At that time, the application process consisted of two phases, a preliminary form (for early identification of projects not deemed compliant with UK Biobank's purposes) and a main form, each requiring separate payment and approval at various levels. This involved reviews from the scientific team to ensure the project was well‐defined and health‐related, the data analysts to ensure the selected data fields were appropriate, UK Biobank's Principal Investigator (UKBPI) to make a final check and the ASC to provide official assessment with approval or rejection (with a right of appeal).

**Figure 2:**
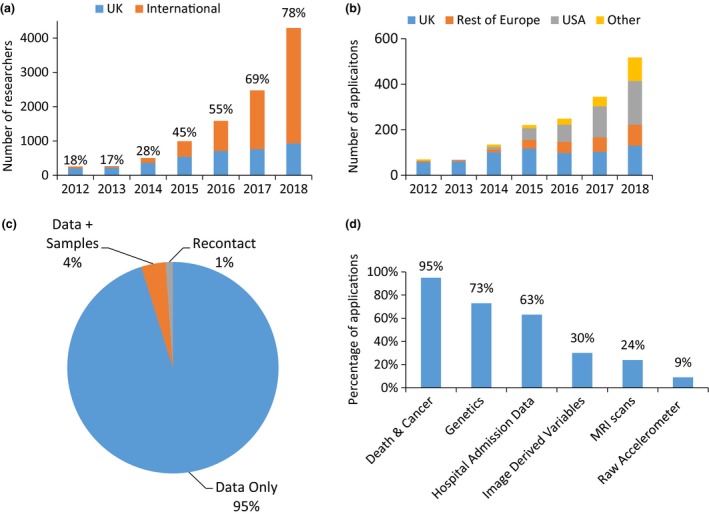
Access metrics. (a) Number of international and UK researchers by year. (b) Number of applications by year and country. (c) Proportion of different types of submitted applications. (d) Proportion of applications requesting different types of data.

Initially, researchers had to have a clear, well‐defined research question with a focus on specific exposures and outcomes and justify their requests for data fields. Data sets were restricted to only those data and participants that the researcher required (e.g. women only or specific case–control subsets). As the sheer size and depth of available data has increased, particularly following inclusion of the genotype data into the resource, the requirements have been relaxed to enable research that is broader in scope and often exploratory in nature (i.e. hypothesis‐generating), with about one‐third of research groups requesting the entire core data set. As interest in the resource has grown over time (see Fig. 2a and b), UK Biobank further streamlined its approach when it launched a new access management system in February 2018 10. Interested researchers still have to register with UK Biobank in order to verify their research credentials, but the application comprises a single simplified form with easier selection of data fields. In a further revision of the process, UK Biobank intends to make it much easier to select the entire core data set (excluding potentially identifying and particularly complex and/or large data) for each research project. It is anticipated this will substantially streamline the process further as it removes the requirement both for researchers to select each data field and for UK Biobank to produce bespoke data sets.

Most data‐only applications are fundamentally noncontentious (with 99% approved), so further streamlining efforts have led to delegation of approval to the scientific team, with the ability to escalate applications to the UKBPI and ASC if considered necessary. These changes have led to a shorter turnaround time for applications: the time from application submission to data release has reduced from 69 weeks in 2013 to 24 weeks by the end of 2018. It is intended that this will continue to be reduced following the changes made to data‐field selection and the removal of an upfront payment stage, to be implemented in mid‐2019.

## Access to biological samples

Applications that request access to biological samples undergo much more stringent consideration, as the samples are a limited and depletable resource. The science behind the request is reviewed rigorously and external expert advice sought, where necessary 11. When the resource was established, it was envisaged that access to the biological samples (blood, urine and saliva) for assays would be coordinated around case–control subsets ‘nested’ within the whole cohort, as performed in virtually all previous prospective studies to date. However, it became apparent that this would not be the most efficient (or cost‐effective) way of developing the resource for researchers to study the causes of many different health outcomes. This is because assays of samples in nested case–control comparisons based on different subsets of the participants preclude reliable comparisons across the full cohort. In contrast, generating assay data from biological samples for the entire cohort at one time facilitates good quality control by reducing measurement error and assay drift. This strategy also minimizes sample depletion and is highly cost‐effective since, in the long term, the costs of conducting assays at one time for all of the participants are likely to be less than the costs of multiple retrievals. As such, requests for UK Biobank samples (which comprise 4% of all submitted applications; Fig. 2c) are now only considered where they are undertaken on the whole (or a large subset) of the cohort, the assay data are applicable to a range of researchers, the assay method is well validated and uses minimal sample volume, and the laboratory can adhere to strict quality control measures 11.

## Access to participants for third‐party studies

At recruitment to the study, participants consented to being re‐contacted by UK Biobank. This includes communications to inform participants about the progress of the study (e.g. via an annual newsletter) and invitations to join third‐party studies. As with samples, UK Biobank considers that re‐contact of participants to be a depletable resource and is mindful not to over‐burden participants with such invitations. Any application to use UK Biobank as a recruitment pool for third‐party studies (which comprise ~1% of all submitted applications; Fig. 2c) is carefully reviewed by the ASC to ensure that there is sufficient scientific justification for such re‐contact. As UK Biobank participants consented on the understanding that no results would be fed back to them following their assessment visits, care is taken to ensure that re‐contact does not represent implicit feedback of information of which participants are not aware. As such, recruitment based on genotype or on phenotype that is not explicitly self‐reported by the participant is highly restricted 12.

## Who is using the data?

**Figure 3:**
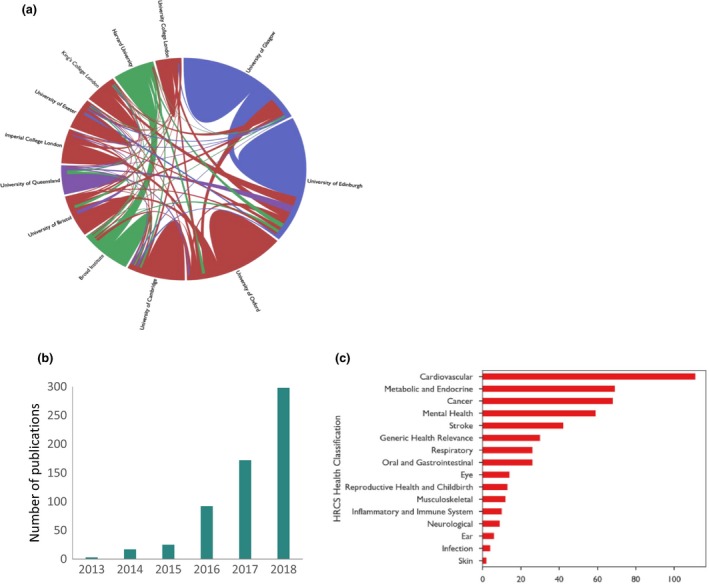
Research metrics. (a) Collaborations between the top 12 institutes (graph generated by Digital Science & Research Solutions Ltd.). (b) Number of publications by year. (c) Areas of research output.

Since 2012, over 10 000 researchers have registered to use the resource, over 1500 applications have been submitted, and 1000 projects are underway. The number of international researchers has steadily increased over time and now accounts for about three‐quarters of all registrations and about two‐thirds of all applications (Fig. 2a and b). Over 700 institutes worldwide have published using UK Biobank data. An independent analysis commissioned in 2018 highlighted that many non‐UK institutes were using the resource with several major international groups – such as the Broad Institute/Harvard (USA), the University of Queensland (Australia), Erasmus University Medical Centre (Netherlands) and the Karolinska Institute (Sweden) – being particularly prolific users. True to the multidisciplinary nature of research, many research groups are collaborating with each other; for example, researchers from the Broad Institute/Harvard and the Universities of Oxford, Cambridge and Edinburgh frequently publish together, as do the Universities of Queensland and Edinburgh (Fig. 3a).

The majority (>95%) of applications are for data‐only (Fig. 2c); true to the prospective nature of the resource, nearly all applications request death and cancer data, approximately three‐quarters request the genomic data, two‐thirds the hospital inpatient data and one‐third the imaging‐derived phenotypes (i.e. variables generated from the raw imaging scans; Fig. 2d).

## Growing interest from industry

The participant consent for UK Biobank is clear that access to the resource is available to commercial companies for use for health‐related research on the same basis as academic researchers. Registered researchers from industry now account for 12% of all researchers as pharmaceutical and other commercial research groups realize the potential of the resource to accelerate drug discovery and develop machine‐learning techniques for early detection of disease. Industry partners are also starting to enhance the resource further (e.g. by supporting cohort‐wide assays) in order to augment their own research aims, whilst at the same time benefiting the wider research community as the enhancements are shared with all researchers after a limited exclusivity period (now set at a fixed period of 9 months).

The first major industry investment was by Regeneron Pharmaceuticals to perform whole exome sequencing of the whole cohort. The first 50 000 samples have been sequenced in partnership with GlaxoSmithKline, and these data are now available to all researchers. The remaining 450 000 samples are being exome sequenced by Regeneron in partnership with Abbvie, Alnylam, AstraZeneca, Biogen, Pfizer, Bristol‐Myers Squibb and Takeda, and will be available to other researchers by the end of 2020. In addition, whole genome sequencing (WGS) is also underway on 50 000 participants, and it is anticipated that sequencing the remaining 450 000 participants will be funded by a consortium of industry, government and charity funders, with data to become available to researchers over the next few years. In parallel, Nightingale Health, a biotech company from Finland, is measuring about 200 lipids and other circulating metabolites for all 500 000 participants. Government and charity funders have also provided funding for academic researchers to measure telomere length for all participants and to collect data on heart arrhythmias via a dedicated heart monitor for 20 000 participants.

In addition, academic and industry collaborations are underway to process the raw scans collected as part of the ongoing imaging assessment of 100 000 participants in order to generate imaging‐derived variables that can be used more readily by the wider research community. Because of the unprecedented scale of the imaging sub‐study, this has necessitated the development of automated processing tools that can rapidly extract imaging‐derived phenotypes. This includes phenotypes related to the structure and function of the brain (developed by The Wellcome Centre for Integrative Neuroimaging 13), liver fat quantity and function (developed by Perspectum 14) and detailed body composition measures (developed by several groups, including Advanced MR Analytics AB in conjunction with Pfizer 15, and Klarismo). These imaging‐derived phenotypes are now being widely used by the wider research community to characterize intermediate disease outcomes and to investigate biological mechanisms of disease.

In this way, industry is effectively becoming a funder of UK Biobank, accelerating the rate at which the biological samples (e.g. through cohort‐wide assays) and complex imaging data (e.g. raw magnetic resonance [MRI] scans) are converted into data that are potentially transformative in terms of the science they can support. Such large‐scale investment is not feasible from most public sector sources, underscoring the effectiveness of UK Biobank's data sharing model.

## Research output

The UK Biobank resource is generating an increasingly large and diverse research output related to identifying genetic and environmental risk factors for disease, with over 600 publications (Fig. 3b) and over 10 000 citations (mostly in the last 2 years), as well as large numbers of conference abstracts, student projects and methodological tools posted online.

The availability of genomic data on such large numbers is transforming genetic research, with genome‐wide association studies (GWAS) now considered routine. Indeed, research groups have already made summary GWAS statistics for thousands of phenotypic traits publicly available 16, 17, 18. This, in turn, is accelerating research into using genetic variants to assess causality of associations (e.g. using Mendelian Randomization approaches 19, 20, 21) or for risk stratification purposes (e.g. using polygenic risk scores 22, 23, 24, 25). For the imaging research community, where MRI data on this scale are unprecedented, both methodological and analytical advancements are underway to maximize the scientific utility of these data. For example, machine‐learning applications are being used to perform segmentation of MRI scans and to predict health outcomes 26.

Linkage to health data is allowing prospective analyses to be undertaken 27, 28, 29, and as the cohort continues to mature, longitudinal research into the causes of a wide range of health outcomes will be possible. To date, cardiovascular, metabolic disease and cancer are the most common outcomes of research interest (Fig. 3c). However, this may well change as the numbers of incident cases of rarer conditions accrue over time. For example, 3000 and 6000 incident cases of osteoarthritis and hip fracture, respectively, will become available by 2022, enabling unprecedented research into their aetiology and progression (Table 1). In addition, the availability of primary care data in UK Biobank – which has hitherto not been available to UK cohort studies at a national level – will facilitate research into conditions (such as asthma, headaches, allergies, back pain, arthritis and diabetes) that are substantially under‐ascertained when based only on hospital admission data. For example, the incorporation of primary care data in UK Biobank is anticipated to more than double the numbers of incident cases of chronic obstructive pulmonary disease (COPD) and dementia compared with hospital records and death data alone.

## Data protection and de‐identification

The processing and use of participant data are heavily regulated activities, particularly following the introduction of the General Data Protection Regulation (GDPR) in May 2018. This resulted in a specific communication to participants 30 setting out how the data that they had provided to UK Biobank were being used in accordance with the GDPR. Participant data provided to researchers are de‐identified, so that potentially identifying information is either not released (e.g. name, NHS number) or is modified (i.e. home location grid coordinates are rounded to 1 km; date of birth is restricted to month and year; certain brain images have facial features removed). UK Biobank is the only party that holds the necessary de‐encryption keys to undertake re‐identification, and different identifiers are used across different UK Biobank internal databases to protect against inappropriate re‐identification (e.g. identifiable information is stored separately from phenotypic and genetic information; data collected during the imaging assessment have different identifiers to those of other data). Access to the keys that link the databases is highly restricted to designated staff to ensure the security of any identifiable data. Additionally, researchers agree when they sign the MTA prior to obtaining the data not to attempt to undertake re‐identification of any participants for any purpose.

UK Biobank has a withdrawal process which allows a participant to withdraw from the resource at any time for any, or indeed no, reason. To date, since recruitment started, fewer than 800 participants have asked to be removed from future data collection (including linkage to electronic health records) and fewer than 200 have asked for their data and samples to no longer be available for research purposes.

## Future direction: dissemination of data

The growing volume of data associated with the increasing richness of the UK Biobank resource will inevitably drive changes in the way those data are disseminated. Hitherto, the approach to data distribution has involved researchers downloading data to their own local computing environment. This has already proved challenging in certain cases. For example, to ensure access for all researchers at exactly the same time, the genotyping data were initially made available in encrypted form and then de‐encrypted simultaneously only when all researchers had had the opportunity to download them (so as not to disadvantage researchers with slower download capabilities).

The sheer volume of data associated with whole exome and whole genome sequencing of the entire cohort (currently estimated to be ~1 PB and ~15 PB, respectively) render unsustainable any approach based on distribution of data to researchers. UK Biobank is already starting to explore platform‐based approaches, bringing researchers to the data rather than sending the data to researchers. By providing access to platforms which allow researchers to use the tools provided by the platform itself, or to run their own tools on the platform, the need to transfer data in bulk is avoided. Such a platform approach may also facilitate use of the UK Biobank resource by researchers at institutions that do not have a significant investment in local IT facilities, thus democratizing further access to the data.

## Conclusion

UK Biobank is being used by thousands of researchers worldwide for health‐related research that is in the public interest. Its open‐access strategy has enabled international scientists to produce excellent science and has led to external investment in enhancing the resource. As global interest in the resource grows, the data access process continues to be streamlined to enable researchers to obtain data quickly and easily. Open access of data to all researchers worldwide has encouraged both public and private investment, thereby enhancing this unique resource further.

## Competing Interests

All authors are current members of UK Biobank scientific team and/or executive management team. All authors have no conflicts of interest to declare.
